# Subclavian vein ultrasound-guided fluid management to prevent post-spinal anesthetic hypotension during cesarean delivery: a randomized controlled trial

**DOI:** 10.1186/s12871-023-02242-6

**Published:** 2023-08-24

**Authors:** Yan Lu, Yueqi Zhang, Zhendong Xu, Fuyi Shen, Jian Wang, Zhiqiang Liu

**Affiliations:** 1 Department of Anesthesiology, Shanghai First Maternity and Infant Hospital, School of Medicine, Tongji University, Shanghai, 200092 China; 2 Department of Anesthesiology, Shuguang Hospital Affiliated With Shanghai University of Traditional Chinese Medicine, Shanghai, China

**Keywords:** Hypotension, Spinal anesthesia, Subclavian vein, Ultrasonography, Cesarean delivery

## Abstract

**Background:**

Hypotension frequently occurs after spinal anesthesia during cesarean delivery, and fluid loading is recommended for its prevention. We evaluated the efficacy of subclavian vein (SCV) ultrasound (US)-guided volume optimization in preventing hypotension after spinal anesthesia during cesarean delivery.

**Methods:**

This randomized controlled study included 80 consecutive full-term parturients scheduled for cesarean delivery under spinal anesthesia. The women were randomly divided into the SCVUS group, with SCVUS analysis before spinal anesthesia with SCVUS-guided volume management, and the control group without SCVUS assessment. The SCVUS group received 3 mL/kg crystalloid fluid challenges repeatedly within 3 min with a 1-min interval based on the SCV collapsibility index (SCVCI), while the control group received a fixed dose (10 mL/kg). Incidence of post-spinal anesthetic hypotension was the primary outcome. Total fluid volume, vasopressor dosage, changes in hemodynamic parameters, maternal adverse effects, and neonatal status were secondary outcomes.

**Results:**

The total fluid volume was significantly higher in the control group than in the SCVUS group (690 [650–757.5] vs. 160 [80–360] mL, p < 0.001), while the phenylephrine dose (0 [0–40] vs. 0 [0–30] µg, p = 0.276) and incidence of post-spinal anesthetic hypotension (65% vs. 60%, p = 0.950) were comparable between both the groups. The incidence of maternal adverse effects, including nausea/vomiting and bradycardia (12.5% vs. 17.5%, p = 0.531 and 7.5% vs. 5%, p = 1.00, respectively), and neonatal outcomes (Apgar scores) were comparable between the groups. SCVCI correlated with the amount of fluid administered (R = 0.885, p < 0.001).

**Conclusions:**

SCVUS-guided volume management did not ameliorate post-spinal anesthetic hypotension but reduced the volume of the preload required before spinal anesthesia. Reducing preload volume did not increase the incidence of maternal and neonatal adverse effects nor did it increase the total vasopressor dose. Moreover, reducing preload volume could relieve the heart burden of parturients, which has high clinical significance.

**Clinical trial registration:**

The trial was registered with the Chinese Clinical Trial Registry at chictr.org.cn (registration number, ChiCTR2100055050) on December 31, 2021.

**Supplementary Information:**

The online version contains supplementary material available at 10.1186/s12871-023-02242-6.

## Background

Most cesarean deliveries are performed under spinal anesthesia. However, spinal anesthesia is responsible for a 70% incidence of hypotension [1]. The high incidence of hypotension can cause adverse effects, including nausea, vomiting, dizziness, fetal acidosis, and hypoxia [2, 3]. To optimize maternal hemodynamics and fetal outcomes, preventive empirical fluid loading is usually performed in obstetric anesthesia before administering a local anesthetic. However, this can lead to volume overload, which is particularly dangerous for parturients with cardiac disease. Thus, optimal fluid administration during cesarean delivery remains unclear [4]. Different techniques, such as esophageal Doppler monitoring or arterial pressure pulse contour analysis, have been described to assess preload in other hemodynamic status factors [5, 6]. However, the widespread use of these techniques remains a subject of ongoing debate owing to financial constraints, relatively high incidence of complications, and potential invasiveness for parturients undergoing cesarean delivery.

Ultrasonography of the inferior vena cava (IVC) in spontaneously breathing patients is recommended as a non-invasive method for estimating the volume status [7]. Measurement of the IVC diameter and its collapsibility index (IVCCI) before spinal anesthesia has been suggested as a method to guide fluid management for preventing hypotension after spinal anesthesia [8]. However, there are limitations to IVC assessment in parturients with enlarged uteri. Previous studies have identified the ultrasonographic determination of the subclavian vein (SCV) collapsibility index (SCVCI) as a rational adjunct to IVCCI in the surgical intensive care unit patient population [9, 10]. It takes less time to acquire SCVCI measurements than IVCCI measurements. Moreover, operators can practice and master this method even if they lack experience in echocardiography. A recent study indicated that pre-anesthetic ultrasonography of the SCVCI could predict hypotension after inducing general anesthesia [11]. However, this has not been studied in patients undergoing spinal anesthesia.

We hypothesized that compared with empirical fluid therapy, SCVCI-guided volume optimization could prevent hypotension after spinal anesthesia in parturients undergoing cesarean delivery. Accordingly, we evaluated the efficacy of SCV ultrasound-guided volume optimization in preventing hypotension after spinal anesthesia during cesarean delivery.

## Materials and methods

### Participants and Group Allocation

This study was approved by the Ethical Committee of Shanghai First Maternity and Infant Hospital (Ethical Committee No. KS21294, approval date: September 28, 2021). All participants provided their written informed consent before the trial. The trial was registered with the Chinese Clinical Trial Registry at chictr.org.cn (registration number, ChiCTR2100055050) on December 31, 2021.

**Fig. 1:**
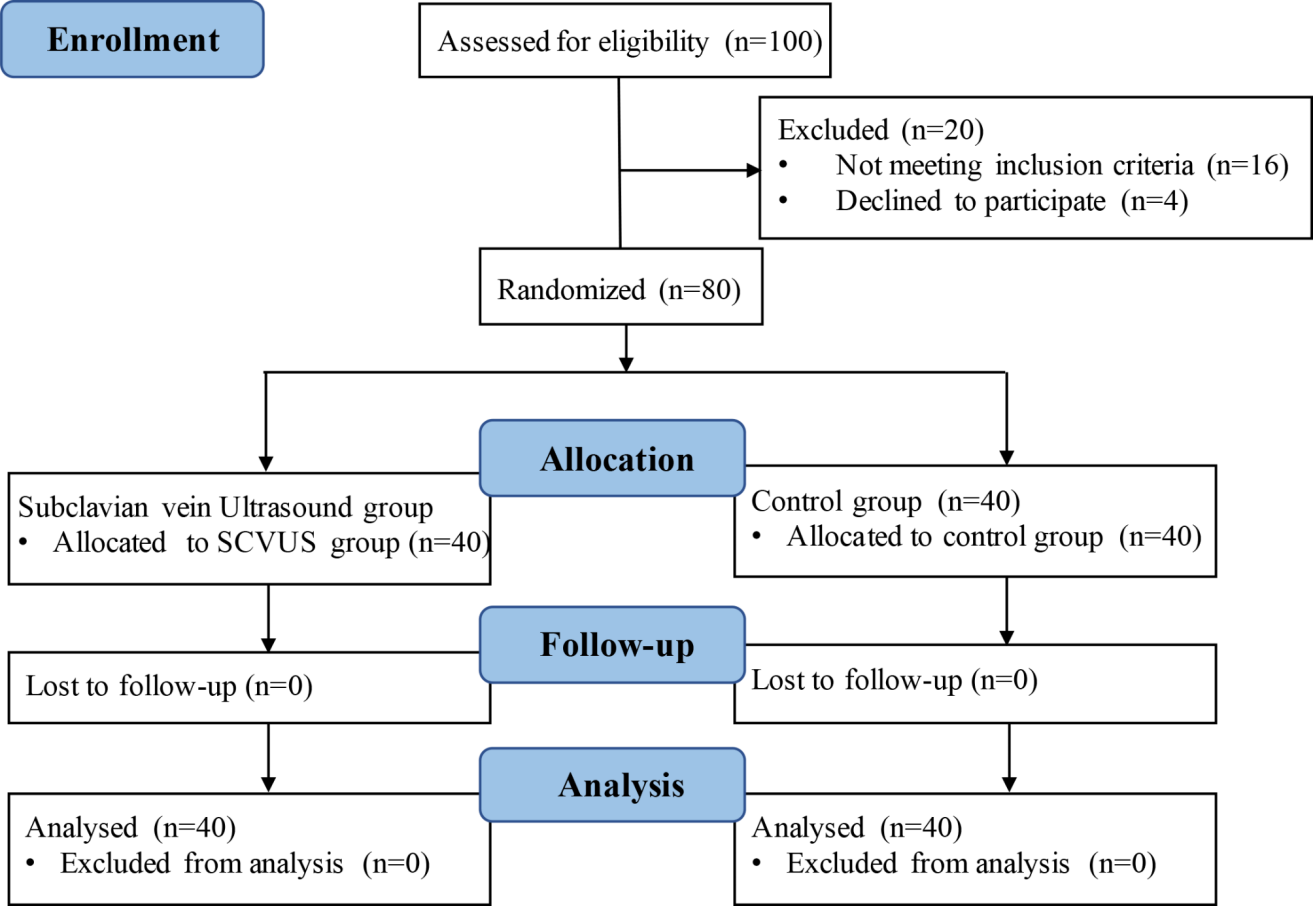
Flowchart of patient enrollment (according to the CONSORT statement)

Eighty term parturients aged 18 to 40 years without any medical or obstetric complications planning for elective cesarean delivery under spinal anesthesia were included in this study. For subject randomization, the statistician employed a computer-generated list they produced before the trial started. Parturients admitted to were recruited from January, 2022, to March. Data have been reported according to the CONSORT guidelines and the enrollment flowchart is presented in Fig. 1. The exclusion criteria were as follows: refusal to participate, contraindications for spinal anesthesia, pre-existing/pregnancy-induced hypertension, known cardiovascular/cerebrovascular diseases, gestational age less than 36 weeks, or baseline systolic blood pressure less than 90 mmHg.

### Hemodynamic monitoring and fluid protocols

Pre-operative fasting was initiated 8 h before surgery, and water intake was permitted until 2 h before surgery. Due to insufficient time for subclavian vein ultrasonography (SCVUS) during spinal anesthesia (the co-loading protocol), we conducted a SCVUS-based preloading protocol (SCVUS group) and compared it with the empirical fixed-volume fluid preload approach (control group).

Upon arrival in the operating room, standard non-invasive anesthesia monitoring, including continuous ECG, non-invasive blood pressure measurements, and oxygen saturation of the pulse (Infinity C500; Dräger Medical, Lübeck, Germany) was started and an 18-G intravenous line was placed. Before implementation of the fluid protocol, patients lay supine in a calm atmosphere for at least 5 min to achieve the proper hemodynamic condition. Treatment allocation was performed by a nurse. Anesthesiologists, researchers, and study participants were blinded to allocation. Subsequently, either the fixed-volume preloading or SCVUS-directed procedure was started before spinal anesthesia by another anesthesiologist who did not take part in anesthetic care.

A single anesthesiologist who was properly trained in ultrasonography performed all SCV measurements with patients in the supine position using GE ultrasonography equipment (GE Medical Systems Ultrasound & Primary Care Diagnostics LLC) before proceeding to spinal anesthesia.

**Fig. 2:**
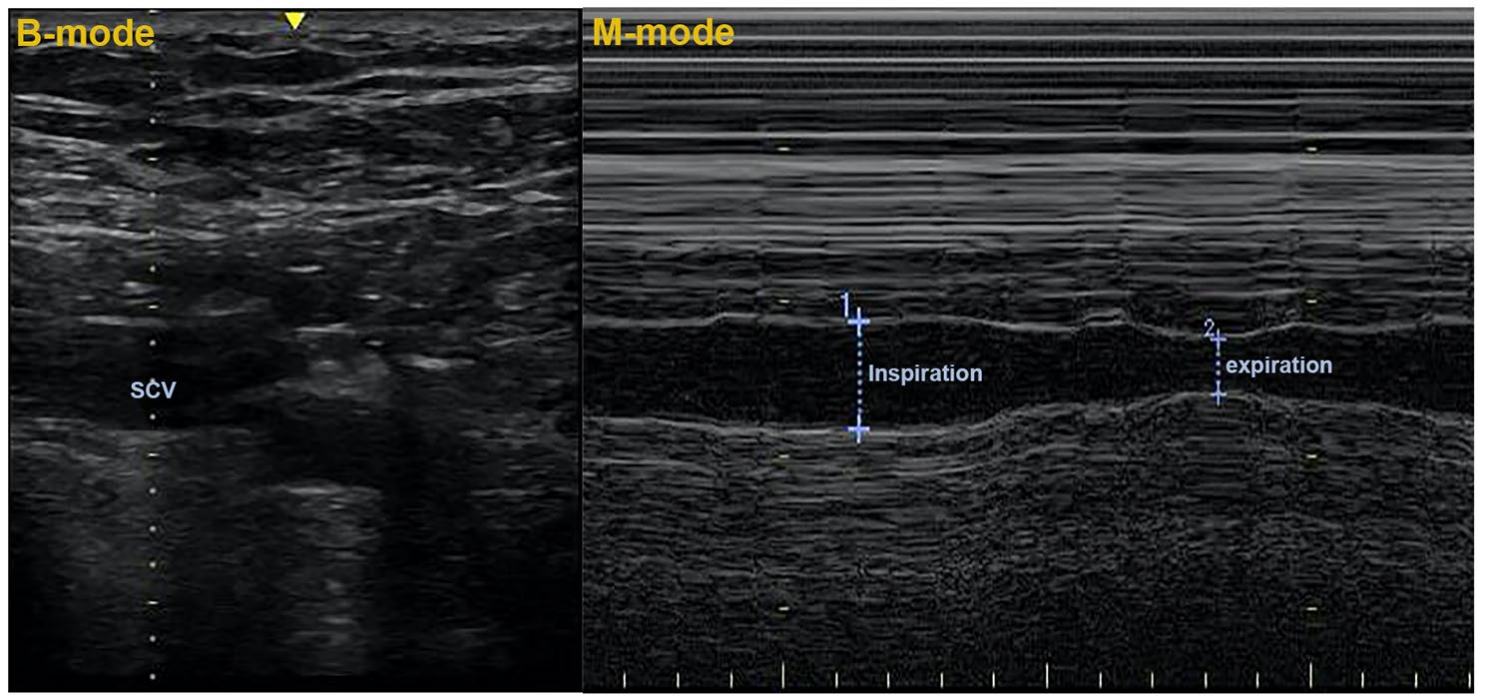
B-mode and M-mode ultrasonography to evaluate SCV with the subcostal view. The SCVCI was calculated as follows: SCVCI=(dSCVmax–dSCVmin)/dSCVmax×100%. The result therefore is expressed as percentages. SCV, subclavian vein; SCVCI, subclavian vein collapsibility index; dSCVmax and dSCVmin, the maximum and minimum antero-posterior diameters of the subclavian vein

Right SCV diameters were measured using an M-mode high-frequency (6–13 MHz) linear array probe. In order to get the best cross-sectional picture of the vein, the probe was positioned beneath the proximal region of the midpoint of the clavicle perpendicular to the long axis of the SCV [12]. The dynamic change in diameter was recorded over time utilizing the M-mode to locate and quantify the minimum and maximum venous diameter during the breathing cycle once the target vein was located (Fig. 2). Three scans were obtained for each patient. The maximum (dSCVmax) and minimum (dSCVmin) antero-posterior diameters of the SCV at the end of the expiration and inspiration periods were recorded during the same respiratory cycle. The SCVCI was calculated using the following formula: SCVCI = (dSCVmax – dSCVmin)/dSCVmax × 100% [9].

A quick infusion of Ringer’s solution (10 mL/kg) was administered to women in the control group within 15 min. Women in the SCVUS group with SCVCI > 38% were considered to have a positive fluid response [8, 9]. Fluid responders were administered a customized fluid treatment regimen that comprised injecting Ringer’s solution (3 mL/kg) over the course of 3 min before re-evaluating the fluctuation in SCV diameter [13, 14]. Similar fluid boluses were administered until a SCVUS non-fluid responder pattern was seen. Subsequently, spinal anesthesia was induced.

### Anesthesia management

Before the implementation of the fluid protocol, the mean of three consecutive measurements was used to calculate the baseline blood pressure, which was taken non-invasively every minute in the arm while the patient remained supine. After the completion of the fluid protocol, patients were subsequently put in a lateral decubitus posture, and spinal anesthesia was induced by inserting a 25-G Whitacre needle (BD Medical, Franklin Lakes, NJ) via an introducer at the L3 or L4 level following skin infiltration with 2–3 mL of 1% lidocaine. After the free flow of cerebrospinal fluid was confirmed, 15 mg of 0.5% isobaric ropivacaine (Naropin, AstraZeneca AB, Södertälje, Sweden) was administered intrathecally. This was done to suppress the sensation of cold and touch at the T6 level. Before skin incision, all patients had a left uterine displacement. Patients who required a rescue epidural dosage or conversion to general anesthesia due to insufficient sensory block or failed spinal anesthesia were not included in the study.

### Management of hypotension after spinal anesthesia

Systolic blood pressure lower than 80% of the baseline after spinal anesthesia was considered hypotension [15] and was treated with 40 µg of intermittent intravenous bolus injections of phenylephrine. If hypotension persisted after two doses of intravenous phenylephrine (80 µg), 100 mL of Ringer’s solution was swiftly administered with an extra dose of phenylephrine. In case of hypotension with a systolic blood pressure drop > 20% of the baseline, 10 µg norepinephrine with 100 mL Ringer’s solution was immediately administered intravenously. An intravenous injection of 0.5 mg atropine was used to treat bradycardia with a heart rate of fewer than 60 beats per minute.

The incidence of post-spinal anesthetic hypotension and maternal side effects, such as nausea, vomiting, bradycardia, and doses of phenylephrine, were noted prior to delivery. Apgar scores were evaluated at 1 and 5 min after delivery.

### Statistical analysis

The primary outcome was the incidence of post-spinal anesthetic hypotension. According to previous studies, the incidence of post-spinal anesthetic hypotension may be as high as 70% [15]. A previous study has also reported that goal-directed fluid management may reduce the incidence of post-spinal anesthetic hypotension by 40% [16]. Thus, after calculation, a sample of 29 patients per group was required to obtain α (type I error) = 0.05 and β (type II error) = 0.1. Forty patients were recruited for each group to account for potential dropouts.

Total fluid volume, vasopressor dosage, changes in hemodynamic parameters, maternal adverse effects, and neonatal status were the secondary outcome measures.

SPSS version 22 (IBM Corp., Armonk, NY, USA) was used to perform statistical analyses. To examine the normality of the distribution of univariate inter-group data, Q-Q plots and the Shapiro–Wilk test were utilized. Normally distributed outcome data are reported as mean (standard deviation [SD]) and Student’s t-test was used to compare groups. The Mann–Whitney U-test was used to examine non-normally distributed data that are summarized as median [interquartile range]. The chi-squared test was used to assess categorical variables. Statistical significance was set at p < 0.05.

## Results

### Characteristics of the participants

The flow of patient recruitment is depicted in Fig. 1. Among 100 women screened for the study, 20 were excluded according to the exclusion criteria and 80 were eventually enrolled for further study. Thus, 40 patients were randomized to the SCVUS group and 40 to the control group. The characteristics of the patients in the two groups were comparable (Table 1).

**Table 1:** Patient characteristics

Variables	Control group (n = 40)	SCVUS group (n = 40)	*p* value
Age (years)	32.35 (4.01)	31.70 (3.31)	0.431
Gestational age (days)	272.45 (6.90)	271.23 (5.37)	0.378
Height (cm)	159.90 (4.43)	160.62 (5.13)	0.505
Weight (kg)	66.08 (8.57)	68.07 (7.68)	0.277
BMI (kg.m^− 2^)	25.71 (2.99)	26.38 (2.65)	0.292
Nulliparous (n)	21	21	1.000

Data are presented as mean (SD) or number

BMI, body mass index; SCVUS, subclavian vein ultrasonography; SD, standard deviation

### Primary outcome

The overall incidence of hypotension after spinal anesthesia was 62.5%. There was no statistically significant difference in the incidence between the SCVUS and control groups (26 patients vs. 24 patients, 65% vs. 60%; p = 0.644). The anesthetic block level in both groups was achieved between T6 and T4, with no difference in the block level between the two groups. All spinal anesthetics were successful (Table 2).

**Table 2:** Intraoperative maternal profiles

	Control group (n = 40)	SCVUS group (n = 40)	*p* value
Fluid protocol time (min)	12 [11–14.5]	11 [6.25–14]	0.069
Pre-anesthesia fluid amount (mL)	600 [550–677.5]	90 [0–287.5]	< 0.001
Pre-anesthesia fluid amount (mL)^a^	600 (500–1060)	90 (0-580)	< 0.001
Total fluid amount (mL)	690 [650–757.5]	160 [80–360]	< 0.001
Sensory blockade			
T6 (n, %)	31 (77.5%)	25 (62.5%)	
T5 (n, %)	1 (2.5%)	1 (2.5%)	0.316
T4 (n, %)	8 (20%)	14 (35%)	
Spinal to delivery time (min)	14 [12–15.75]	14 [12–16]	0.950
Post-spinal anesthetic hypotension (n, %)	26 (65%)	24 (60%)	0.644
Phenylephrine dose (µg)	0 [0–40]	0 [0–30]	0.276
Nausea and/or vomiting (n, %)	5 (12.5%)	7 (17.5%)	0.531
Bradycardia (n, %)	3 (7.5%)	2 (5%)	1.000

Data are presented as median [interquartile range] or number (%)

^a^ Data are presented as median (min-max)

SCVUS, subclavian vein ultrasonography

### Secondary outcome

A significant difference in the volume of pre-anesthesia-administered fluid was found between the two groups (600 mL in the control group vs. 90 mL in the SCVUS group, p < 0.001). Similarly, the amount of total fluid administered was 690 mL in the control group and 160 mL in the SCVUS group (p < 0.001). Phenylephrine dose requirements were comparable between the two groups (p = 0.276). Women in both groups demonstrated similar negative effects associated with spinal anesthesia, including bradycardia, nausea, and/or vomiting (Table 2).

### Neonatal outcome

The neonatal outcomes are summarized in Table 3. The 1-min and 5-min Apgar scores were comparable between the groups.

**Table 3:** Neonatal profiles

	Control group (n = 40)	SCVUS group (n = 40)	*p* value
Neonatal Apgar score			
1 min	9 (9–10)	9 (8–10)	0.482
5 min	10 (9–10)	10 (9–10)	0.559

Data are presented as median (range)

SCVUS, subclavian vein ultrasonography

### Correlation of SCVCI Value with Pre-anesthesia Fluid volume

**Fig. 3:**
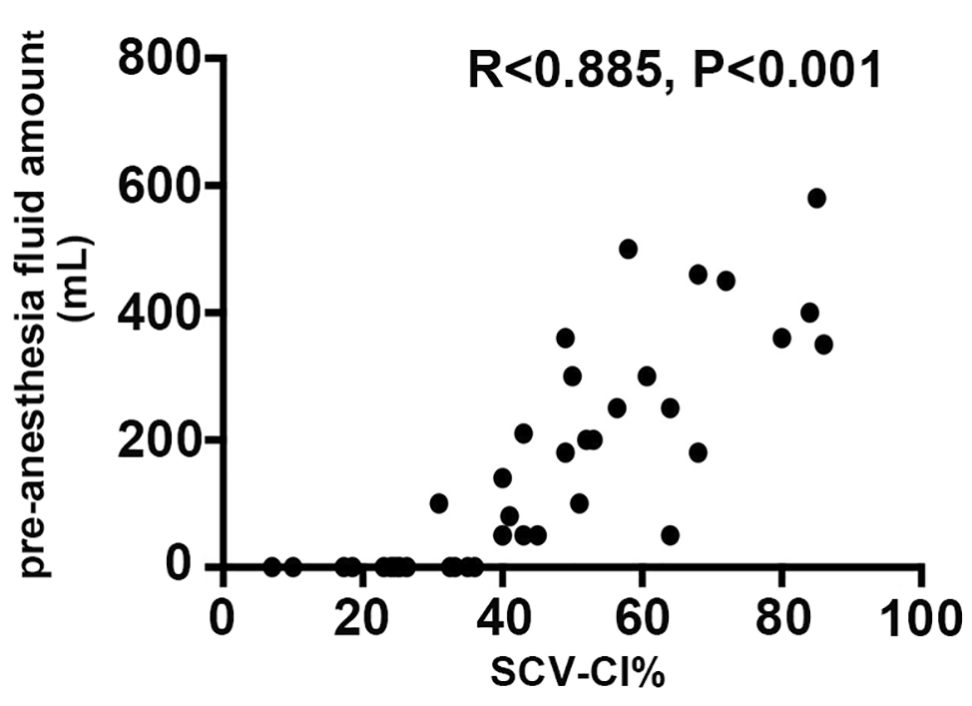
Correlation analysis between SCVCI (expressed as a percentage) and pre-anesthesia fluid amount (expressed in mL). This correlation was probably explained in part by the study design. An increase in SCVCI is correlated with an administration of fluid. SCVCI, subclavian vein collapsibility index

There was a positive correlation between the SCVCI value and the pre-anesthesia fluid volume (R = 0.885, p < 0.001) (Fig. 3). When the SCVCI value increased, the pre-anesthesia fluid volume was ramping up.

## Discussion

This randomized controlled trial failed to demonstrate a significant difference in the incidence of hypotension after spinal anesthesia for elective cesarean delivery when using the SCVUS-based preloading protocol and the conventional fixed-volume fluid preload protocol. Although using SCVUS for patient-customized fluid treatment before spinal anesthesia during cesarean delivery did not lead to a decrease in the incidence of spinal anesthesia-induced hypotension, we observed a significant reduction in fluid preload when using the SCVUS-based preloading protocol. Moreover, reducing fluid preload neither increased the incidence of maternal and neonatal adverse effects nor increased the total vasopressor dose.

Hypotension following spinal anesthesia for cesarean delivery has been thought to develop due to venous vasodilation; therefore, for many years, fluid-loading strategies have remained a major part of the anti-hypotensive strategy. A status of sufficient volume loading may reverse relative hypovolemia due to vasodilation and help maintain hemodynamic stability. However, there is still no consensus on fluid-loading strategies for obstetric patients. This study showed that the fixed-volume preload strategy recommended by the British guidelines (2011) [17] and the American Society of Anesthesiologists/Society for Obstetric Anesthesia and Perinatology Task Force 2016 [18] was not effective in improving post-spinal anesthetic hypotension. A recent meta-analysis showed that in 109 trials and 12 analysis methods, fixed-volume crystal preload was the most common method to prevent hypotension after cesarean delivery [3]. However, it has been discovered that preloading with crystalloid fluids prior to spinal anesthesia is not useful in preventing hypotension [19]. In our study, SCVUS-guided fluid preload did not reduce the incidence of spinal-anesthesia-induced hypotension but significantly decreased the amount of fluid preload, which may be more beneficial for the obstetric population. Pregnancy causes a rise in blood volume, which increases the amount of blood returning to the heart (preload) [20]. Excessive fluid preload increases the burden on the heart, especially in pregnant women with cardiac insufficiency, causing adverse consequences. Although the amount of fluid preload decreased significantly in the SCVUS group, the incidence of nausea and vomiting was similar between the groups. However, none of the strategies adopted in this study could prevent hypotension after spinal anesthesia. A previous study on maternal cardiac output during cesarean delivery showed that arterial vasodilation might be more likely to contribute to the drop in blood pressure after spinal anesthesia [21].

Good neonatal outcomes, as indicated by Apgar scores, were also similar in the two groups. We did not measure umbilical cord gases, as previous studies have demonstrated that fluid strategy does not influence neonatal acid-base status when maternal hypotension is treated [22, 23].

Ultrasonography has been used in adults as a rapid and objective tool for assessing intravascular status [24]. Previous research has demonstrated that measuring the IVC diameter using ultrasound is helpful for assessing intravascular volume status of patients [25]. However, ultrasonographic measurement of the IVC diameter may be limited by an enlarged uterus in obstetric patients. A recent study has shown that ultrasonographic measurement of the SCV appears to be a reasonable replacement for IVC in the surgical intensive care unit patient population. The overall measurement bias is minimal, and the correlation between the two methods is satisfactory. Additionally, SCVCI measurements require less time than IVCCI measurements, though it is unclear how this affects clinical outcomes [9]. In this study, we identified fluid responders using an SCVCI value > 38% and performed volume optimization before spinal anesthesia. In a study on fluid responsiveness and SCV collapsibility in ICU patients on mechanical ventilation, collapsibility values of responders, whose cardiac output improved by > 15% following fluid challenge, and non-responders were 27.9 ± 14.4% and 8.5 ± 4.9%, respectively [26]. The cut-off values of IVCCI vary across studies, and have been reported as 36% [8] and 42% [27]. In a study comparing SCVCI and IVCCI to evaluate the intravascular volume status in ICU patients, the IVCCI was 23.5 ± 15.2% and SCVCI was 26.7 ± 17.8% [9]. We also found a positive correlation between the SCVCI value and the pre-anesthesia fluid volume (R = 0.885, p < 0.001) in our study. With an increase in the rehydration volume, the SCVCI value decreased, indicating that the use of SCVCI was a good method to evaluate the intravascular volume status. Because the anatomical position is fixed, SCVCI evaluation has clavicle support to avoid measurement failure due to compression and deformation during the measurement process, and is not affected by abdominal pain, obesity, or pregnancy. In emergent events, ultrasound monitoring of SCV is not limited by the surgical area disinfection and is easily accessible. Therefore, the SCVCI evaluation may be inferior to the IVCCI evaluation in the event of an emergency [28].

This study had some limitations. First, the sample size was relatively small. Furthermore, we only included relatively stable patients to test our hypothesis, which may have affected the generalizability of this SCVCI index in special cases. Second, all ultrasonographic measurements of SVC diameter were performed by an anesthesiologist fully trained in ultrasonography, which might restrict the applicability of our findings in situations when ultrasonic exams are carried out by untrained doctors. Third, patients that receive more fluid may respond with higher urinary output. We didn’t measure the relationship between urine output with rehydration volume in our study. A previous study demonstrated that there was no association between increased fluid volume administration and augmented urinary output under general anesthesia [29], and further studies are needed to explore the relationship between rehydration volume and urine output under spinal anesthesia. Finally, we only measured the SCVCI during normal spontaneous breathing and did not obtain corresponding data during deep breathing, which should be validated in future studies.

In conclusion, SCVUS-guided volume management did not ameliorate post-spinal anesthetic hypotension but reduced the volume of fluid preload required before spinal anesthesia. Reducing preload volume did not increase the incidence of maternal and neonatal adverse effects nor did it increase the total vasopressor dose. More importantly, reducing preload volume could relieve the heart burden of parturients, which has high clinical significance.

Future studies should focus on the best practice of the combination of fluid-loading strategies and prophylactic use of vasopressors to prevent post-spinal anesthetic hypotension.

### Electronic supplementary material

Below is the link to the electronic supplementary material.

Supplementary Material 1

## Data Availability

The datasets presented in this study are included in the article, further inquiries can be directed to the corresponding authors.

## Abbreviations

dSCVmax: maximum antero-posterior diameter of the subclavian vein
dSCVmin: minimum antero-posterior diameter of the subclavian vein
IVC: inferior vena cava
IVCCI: inferior vena cava collapsibility index
SCV: subclavian vein
SCVCI: subclavian vein collapsibility index
SCVUS: subclavian vein ultrasonography
