# Supplementary material for: Subclavian vein ultrasound-guided fluid management to prevent post-spinal anesthetic hypotension during cesarean delivery: a randomized controlled trial
Source: BMC Anesthesiol. 2023 Aug 24;23:288. doi: 10.1186/s12871-023-02242-6 (PMC10464078; doi:10.1186/s12871-023-02242-6)
Supplement: Supplementary file 1 — Supplementary Material 1 [file 12871_2023_2242_MOESM1_ESM.docx]

supplementary material

|  | **Control group**  **(n=40)** | **SCVUS group**  **(n=40)** | ***p* value** |
| --- | --- | --- | --- |
| Baseline systolic pressure (mmHg) | 119.43 (8.71) | 115.9 (8.55) | 0.071 |
| Baseline HR (beats/min) | 78.25 (10) | 81.93 (9.58) | 0.097 |
| Lowest systolic pressure before delivery (mmHg) | 93.15 (14.96) | 88.23 (17.64) | 0.182 |

Data are presented as mean (SD).

Legend: maternal baseline systolic pressure and heart beat before anaesthesia induction, and the lowest maternal systolic pressure before delivery.
